# Hypertensive crisis caused by electrocauterization of the adrenal gland during hepatectomy

**DOI:** 10.1186/1471-2482-15-11

**Published:** 2015-02-14

**Authors:** A Ram Doo, Ji-Seon Son, Young-Jin Han, Hee Chul Yu, Seonghoon Ko

**Affiliations:** Department of Anesthesiology and Pain Medicine, Research Institute of Clinical Medicine, Chonbuk National University Medical School and Hospital, Jeonju, Republic of Korea; Department of Surgery and Research Institute of Clinical Medicine, Chonbuk National University Medical School and Hospital, Jeonju, Republic of Korea

**Keywords:** Adrenal gland, Electrocautery, Hepatectomy, Hypertensive crisis

## Abstract

**Background:**

Hypertensive crisis (i.e., systolic blood pressure over 300 mmHg) is very rare during operation except pheochromocytoma, but it can be a fatal and embarrassing to surgeons and anesthesiologists. The right adrenal gland can be electrocauterized during a right hemi-hepatectomy. We report a case of hypertensive crisis during right hemi-hepatectomy in which the right adrenal gland was stimulated by monopolar electrocautery in a patient with normal neuroendocrine function.

**Case presentation:**

A 73-year-old man with hepatocellular carcinoma was scheduled to undergo right hemi-hepatectomy. Three hours into the surgery, the patient’s blood pressure increased abruptly from 100/40 to over 350/130 mmHg (the maximum measurement pressure of the monitor; 350 mmHg). The surgeon had cauterized the right adrenal gland using monopolar electrocautery to separate the liver from the adrenal gland immediately prior to the event. Approximately 3 minutes after suspending the operation, blood pressure returned to baseline levels. After the event, the operation was successfully completed without any complication. Hormonal studies and iodine-123 meta-iodobenzylguanidine scintigraphy revealed no neuroendocrine tumor such as a pheochromocytoma.

**Conclusion:**

Operations such as hepatectomy that stimulate the adrenal gland may lead to an unexpected catecholamine surge and result in hypertensive crisis, even if neuroendocrine function of the adrenal gland is normal.

## Background

Intraoperative hypertension is common during general anesthesia, but it is usually well managed without serious complications. However, severe hypertension can cause cardio-cerebrovascular complications such as myocardial ischemia, arrhythmia, cerebral hemorrhage, or aortic dissection during the perioperative period. Although controlling blood pressure has become easier to accomplish due to the development of anesthetic techniques and antihypertensives, severe hypertension still remains a challenging issue [1, 2]. In particular, severe hypertension associated with secondary causes such as pheochromocytoma, carcinoid syndrome, or thyroid storm are even more difficult to manage during an operation [3–5].

The adrenal gland stores and discharges catecholamines into the blood stream by activation of the sympathetic nervous system. Surgical manipulation of the adrenal gland, especially in the catecholamine producing tumors such as pheochromocytoma and paragangliomas, may cause a catecholamine surge resulting in hypertensive crisis intraoperatively [3, 6]. Furthermore, a case of severe hypertension caused by radiofrequency ablation of a metastatic adrenal gland tumor with normal neuroendocrine function has been reported [7].

We report a case of abrupt and severe hypertension, with systolic blood pressures over 350 mmHg, during a right hemi-hepatectomy in which the normal neuroendocrine functioning adrenal gland was electrically stimulated by monopolar electrocautery to separate the liver from the adrenal gland.

## Case presentation

A 73-year-old man with a 10-year history of hypertension and diabetes mellitus presented with hepatocellular carcinoma and complicated cyst in the liver. The patient was scheduled to undergo right hemi-hepatectomy. Hypertension was controlled with an angiotensin II receptor antagonist. Diabetic neuropathy was diagnosed in autonomic function test. Magnetic resonance imaging of the abdomen revealed a 2.6 × 2.6 sized hepatocellular carcinoma in segment VII and a 3.2 × 3.2 sized atypical complicated cyst in segment VIII. The patient had no abnormal findings in the adrenal gland, spleen, and pancreas, except for a simple renal cyst.

Initial blood pressure was 150/80 mmHg and heart rate was 65 beats per minute in the operating room. Anesthesia was induced with thiopental 300 mg, rocuronium 60 mg, and continuous infusion of remifentanil (effect site concentration 3 ng/ml) using a target controlled infusion system (Orchestra® Primea, Fresenius vial, Brezins, France). After endotracheal intubation, anesthesia was maintained with 1-3% of sevoflurane and remifentanil 2–4 ng/ml effect site concentration. The left radial artery was cannulated for direct blood pressure monitoring using a pressure kit (Tru-Wave® Disposable Pressure Transducer, Edwards Lifesciences, Irvine, USA). The right subclavian vein was catheterized for fluid management and central venous pressure monitoring.

From the start of the operation, blood pressures ranged from 100/40 to 150/70 mmHg with heart rates 55 to 70 beats per minute for three hours. Three hours into the surgery, systolic blood pressure abruptly increased from 100 to over 350 mmHg, the maximum measurable pressure of the monitor incorporated with anesthesia workstation (Zeus® anesthesia machine, Drager AG, Lübeck, Germany). Diastolic blood pressure increased from 40 to 130 mmHg and heart rate increased from 75 to 95 beats per minute. The anesthesiologist checked the delivery system of the anesthetics, which was functioning normally. No drug was injected at that time. Anesthesia was deepened and labetalol 10 mg was administered.

The operation was stopped immediately. Although a vasopressor such as epinephrine was not injected in the surgical field, the right adrenal gland had been cauterized by monopolar electrocautery to separate the liver from the adrenal gland. Approximately 3 minutes after suspending the operation, blood pressure and heart rate returned to baseline levels, which were maintained until the end of the operation. After the event, the right liver was resected successfully without any complication.

Postoperatively, the patient was transferred to the intensive care unit for hemodynamic monitoring and neurologic examination. Neurologic examination performed by a neurologist was within normal limits, and the patient remained hemodynamically stable during recovery. Hormonal studies were performed to investigate an undetected pheochromocytoma or paraganglioma. However, urine metanephrine, vanillylmandelic acid, and homovanillic acid levels were 0.3 mg/day (normal reference: 0–0.8 mg/day), 2.3 mg/day (0–8 mg/day) and 2.8 mg/day (1.4-8.8 mg/day), respectively. Iodine-123 meta-iodobenzylguanidine (^123^I-MIBG) scintigraphy revealed no neuroendocrine tumors such as pheochoromocytoma. The postoperative course was uneventful, and the patient was discharged without any sequelae.

## Conclusion

Severe hypertension is defined as an increase in systolic blood pressure ≥ 180 mmHg or diastolic blood pressure ≥ 110 mmHg without end-organ damage. Hypertensive emergency is defined as rapidly evolving end-organ damage associated with hypertension of diastolic blood pressure > 120 mmHg [8]. Intraoperative hypertensive emergency is rare, but it may cause critical complications such as cerebral hemorrhage, acute congestive heart failure, myocardial infarction or aortic dissection. Rapid reduction of blood pressure is required for better patient outcomes during hypertensive emergency. In our patient, systolic blood pressure was recorded over 350 mmHg. The arterial waveform was cut-off at 350 mmHg, suggesting that the systolic blood pressure was more likely 380 mmHg according to arterial waveform. Although the systolic blood pressure of the patient abruptly peaked over 350 mmHg, this critical moment lasted for only a very short period. The patient had no evidence of end organ damage and recovered without complications.

Paix et al. [9] reported that factors causing intraoperative hypertension include drug-related causes (59%), such as vasopressor administration by the anesthesiologist or surgeon, excessive surgical stimulation or light anesthesia (21%), equipment-related problems (13%), and miscellaneous causes (7%). When considering the possible causes of severe hypertension in the patient, no drug was administrated and anesthetic depth was sufficient for surgical stimulation. The right adrenal gland was electrocauterized just before the episode. Furthermore, this severe hypertension quickly normalized within a few minutes once surgical manipulation was suspended. In this setting, the authors suspected a pheochromocytoma of the right adrenal gland. Pheochromocytomas, neuroendocrine tumors that secrete catecholamines derived from adrenal chromaffin cells, causes secondary hypertension due to uncontrolled release of catecholamines including norepinephrine and epinephrine [6, 10]. Pheochromocytomas usually manifest visible signs and symptoms such as paroxysmal hypertension, headache, sweating, and flushing, but some reports have suggested the possibility of unexpectedly encountering a pheochromocytoma during general or spinal anesthesia for a surgery [11–13]. In this case, catecholamine metabolites including urine metanephrine, vanillylmandelic acid, and homovanillic acid, were within normal ranges and ^123^I-MIBG scintigraphy revealed no neuroendocrine tumor such as a pheochromocytoma or paragalglioma.

Chini et al. [7] reported a case of hypertensive crisis during radiofrequency ablation of adrenal metastasis. They assumed that the hypertensive crisis was related to injury of normal adrenal tissue or the ablated adrenal mass, resulting in systemic catecholamine release. Onik et al. [14] similarly reported that hypertensive crisis during radiofrequency ablation of the liver. Because the postero-inferior portion of the right hepatic lobe is the positional proximity to the right adrenal gland, the manipulation such as electrocauterization of the adrenal gland is inevitable during right hemi-hepatectomy. It is remarkable that a surge of catecholamines can be released from a normal adrenal gland without evidence of a pheochromocytoma. The authors assumed that monopolar electrocauterization stimulated the release of catecholamines stored in the sympathetic postganglionic nerve fibers or the adrenal medulla in this patient. The monopolar electrosurgical unit delivers high-frequency current at the tip of the active electrode producing heat and energy that facilitates cutting, coagulation, and desiccation. Monopolar electrocautery has inherent risks including burns, electromagnetic interference, and unwanted muscle or nerve stimulation. Bipolar electrocautery has lower risk of unintentional spread of electrical currents to the surrounding tissue than monopolar electrocautery [15].

In summary, intraoperative stimulation of the adrenal gland has the potential risk of unexpected catecholamine surge, resulting in severe hypertension even if the neuroendocrine function of the adrenal gland is normal.

## Consent

Written informed consent was obtained from the patient for publication of this Case Report. A copy of the written informed consent is available for review by the Editor of this journal.
